# NHS announces a pilot of a blood test for early detection of many cancers

**DOI:** 10.1177/0969141320986823

**Published:** 2021-02-24

**Authors:** Robert Old, Paul Pharoah, Nicholas Wald

**Affiliations:** 1 School of Life Sciences, Warwick University, Coventry, UK; 2 Department of Public Health and Primary Care, University of Cambridge, Cambridge, UK; 3 Institute of Health Informatics, University College London, London, UK

In November 2020, a public announcement was made of a partnership between NHS England and GRAIL, an American healthcare company that has been developing a plasma DNA-based test called ‘Galleri’ for many cancer sites. Sir Simon Stevens, NHS Chief Executive, has called the blood test potentially game-changing for the early detection of cancers.^1^ The announcement states that “a pilot” “will involve 140,000 participants aged 50 to 79 who have no symptoms but will have annual blood tests for three years”. If this is correct, the application of the test would effectively be considered as a new screening method. The announcement also states that “another 25,000 people with possible cancer symptoms will also be offered testing to speed up their diagnosis after being referred to hospital in the normal way”, implying the introduction of the test into routine medical practice. It is unfortunately unclear precisely what is planned and what are the scientific objectives.

The GRAIL test depends upon finding DNA markers of cancer in peripheral blood. It exploits the fact that tumour formation depends upon DNA mutations acquired during life (somatic mutations), in some cases in combination with inherited mutations. At the cellular level, gain-of-function mutations in proto-oncogenes such as *C-MYC* in many tumour types or *BRAF* in some colon cancers, together with loss-of-function mutations in tumour suppressor genes such as *TP53* and *BRCA1* in breast cancer, combine to disrupt a few key cellular pathways for cell-cycle regulation, cell signalling and DNA metabolism. As malignancy evolves through genomic instability and selection for cell proliferation, these so-called driver mutations accumulate together with less functionally relevant ‘passenger’ mutations. Remarkably, driver mutations in as few as about 160 genes out of the whole genomic complement account for nearly all tumorigenesis. In addition to mutations in particular genes, genomic instability results in changes in the number of copies of DNA regions, either their amplification or deletion, and hypermethylation of the DNA in many regions of the genome. As tumours develop, some altered DNA is released in the form of cell-free fragments, presumably through cell death, into blood. Early in the disease process, the altered DNA is present in plasma in very small amounts. The GRAIL test uses the most sensitive DNA analytical methods to detect these fragments and characterise them. These methods have sometimes, perhaps optimistically, been called a ‘liquid biopsy’ for a range of tumours.

The use of cell-free DNA analysis in screening has great potential, but unlike its use in antenatal screening for Down’s syndrome, trisomy 18 and trisomy 13, its use in cancer screening remains uncertain. Four questions need to be answered before service pilot studies are launched. First, what is the screening performance of the GRAIL blood test for specific cancer sites expressed as detection rates (sensitivity) for specified false-positive rates for given intervals before clinical presentation? In results on DNA methylation in a case-control study, the reported detection rate was only 18% of stage 1 cancers for a 0.7% false-positive rate.^2^ Screening performance can be estimated with the data from a large prospective study adopting a nested case-control design using stored plasma samples. Second, what is the accuracy in identifying the site of the cancer in people with screen positive results among asymptomatic people? Third, what is the probability that the source of the cancer in people with screen positive results cannot be found in diagnostic investigations (e.g. magnetic resonance imaging or endoscopy) or the probability they will find unexpected abnormalities of little or no clinical significance? Fourth, what, in a large randomised trial, is the reduction in the pre-specified site-specific cancer mortality rate (not survival because of lead time and length of time bias, and not cancer incidence which increases in screening due to early detection) following the treatment of people with screen positive results?

The design of the study involving 140,000 people without symptoms (i.e. screening) is unclear from what is publicly available. Once there is clarity over what is planned and the above questions are addressed and published, a pilot study could be considered on many fewer people. A starting point before considering the test for screening would be to assess the test performance in patients with symptomatic stage I tumours for the individual cancer sites the test would be used to screen for. If that shows poor performance, it is likely to be even poorer in asymptomatic patients and may not therefore be worth taking further.

We are concerned that the study design and aims described in the public announcement are not clear, and that the NHS may be taking on a major screening initiative without obtaining the necessary evidence on efficacy, safety and cost to see if they meet the recognised requirements of a worthwhile cancer screening test.^3^
